# Electroacupuncture Ameliorates Mechanical Allodynia of a Rat Model of CRPS-I via Suppressing NLRP3 Inflammasome Activation in Spinal Cord Dorsal Horn Neurons

**DOI:** 10.3389/fncel.2022.826777

**Published:** 2022-05-25

**Authors:** Yunwen Zhang, Ruixiang Chen, Qimiao Hu, Jie Wang, Huimin Nie, Chengyu Yin, Yuanyuan Li, Huina Wei, Boyu Liu, Yan Tai, Junfan Fang, Xiaomei Shao, Xiaoqing Jin, Jianqiao Fang, Boyi Liu

**Affiliations:** ^1^Key Laboratory of Acupuncture and Neurology of Zhejiang Province, Department of Neurobiology and Acupuncture Research, The Third Clinical Medical College, Zhejiang Chinese Medical University, Hangzhou, China; ^2^Centre for Neurodevelopmental and Neurodegenerative Diseases, The Brain Cognition and Brain Disease Institute of Shenzhen Institute of Advanced Technology, Chinese Academy of Sciences, Shenzhen, China; ^3^Academy of Chinese Medical Sciences, Zhejiang Chinese Medical University, Hangzhou, China; ^4^Department of Acupuncture, Zhejiang Hospital, Hangzhou, China

**Keywords:** allodynia, inflammasome, electroacupuncture, spinal cord, glial cell, complex regional pain syndrome

## Abstract

Complex regional pain syndrome type-I (CRPS-I) is a chronic neurological disorder that results in severe pain and affects patients' life quality. Conventional therapies usually lack effectiveness. Electroacupuncture (EA) is an effective physical therapy for relieving CRPS-I pain. However, the mechanism underlying EA-induced analgesia on CRPS-I still remain unknown. Spinal NLRP3 inflammasome was recently identified to contribute to pain and neuroinflammation in a rat model of CRPS-I by our group. Here, we aimed to study whether EA could inhibit spinal NLRP3 inflammasome activation, thus resulting in pain relief and attenuation of spinal neuroinflammation in the rat model of CRPS-I. We established the rat chronic post-ischemic pain (CPIP) model to mimic CRPS-I. CPIP rats developed remarkable mechanical allodynia that could be relieved by daily EA intervention. NLRP3 inflammasome was activated in spinal cord dorsal horn (SCDH) of CPIP rats, accompanied with over-production of pro-inflammatory cytokine IL-1β. Immunostaining revealed that the cellular distribution of NLRP3 was predominantly located in SCDH neurons. Pharmacological activation of NLRP3 inflammasome *per se* is sufficient to produce persistent mechanical allodynia in naïve animals, whereas blocking NLRP3 inflammasome attenuates mechanical allodynia of CPIP rats. EA exclusively reduced NLRP3 overexpression in SCDH neurons and attenuated spinal glial cell over-activation in CPIP rats. EA-induced anti-allodynia with attenuation of spinal glial cell over-activation were all mimicked by intrathecal blocking NLRP3 inflammasome and reversed by activating NLRP3 inflammasome, respectively, through pharmacological methods. Finally, spinal blocking IL-1β attenuated mechanical allodynia and spinal glial cell over-activation in CPIP rats, resembling the effects of EA. In all, these results demonstrate that spinal NLRP3 inflammasome activation contributes to mechanical allodynia of the rat model of CRPS-I and EA ameliorates mechanical allodynia through inhibiting NLRP3 inflammasome activation in SCDH neurons. Our study further supports EA can be used as an effective treatment for CRPS-I.

## Introduction

Complex regional pain syndrome type-I (CRPS-I) is a chronic neurological disorder that severely affects patients (Ott and Maihofner, 2018). It is oftentimes triggered after an initial injury, including ischemia, soft tissue trauma, surgery, or fractures to the extremity (Coderre and Bennett, 2010; Goh et al., 2017; Birklein et al., 2018; Urits et al., 2018). It is characterized with spontaneous pain and thermal/mechanical pain hypersensitivities, accompanied with edema and changes in skin blood flow in the affected extremities (Johnston-Devin et al., 2021). The chronic pain severely affects the patients' life quality, both physically and mentally (Lee et al., 2014; Goh et al., 2017; Johnston-Devin et al., 2021). Certain patients even develop suicidal tendencies due to CRPS (Lee et al., 2014). However, conventional medications, including non-steroidal anti-inflammatory drugs and corticosteroids, etc. usually lack effectiveness on CRPS-I (Kingery, 1997; Bruehl, 2010). Therefore, identifying alternative therapies for the management of CRPS-I-related pain has important clinical significance.

One such potential therapeutic option for CRPS-I-related pain is electroacupuncture (EA). EA is a physical therapeutic method that integrates traditional manual acupuncture with modern electrotherapy. It shows effectiveness on many pain symptoms in clinic (Vickers et al., 2018). Recently, one meta-analysis concluded that EA can effectively alleviate pain and improve daily activities of CRPS-I patients (Wei et al., 2019). This result suggests that EA can be used in clinic for CRPS-I-related pain management. In addition, our recent study confirmed EA's analgesic effect in a rat model of CRPS-I and we further identified 2/100 Hz as an effective parameter for EA interventions (Hu et al., 2020a; Li et al., 2022). Although EA's therapeutic effect on CRPS-I is well-documented, yet the mechanisms contributing to EA's analgesic effects on CPRS-I still remain largely unexplored.

NLRP3 inflammasome is a protein complex consisting of NLRP3/ASC/caspase-1 (Alexander et al., 2019; O'Brien et al., 2020). The activation of NLRP3 inflammasome cleaves pro-IL-1β into active IL-1β (Feng Y. S. et al., 2020). IL-1β has been well-established to be a pivotal pain mediator which induces or maintains pain *via* direct activation or sensitization of nociceptors (Safieh-Garabedian et al., 1995; Binshtok et al., 2008). NLRP3 inflammasome activation in peripheral tissues or sensory nerves is involved in both inflammatory and neuropathic pain (Chen et al., 2021). The expression of NLRP3 inflammasome in spinal cord has been documented (Grace et al., 2016). Our recent study showed that NLRP3 inflammasome is activated in the spinal cord of a rat model of CRPS-I (Chen et al., 2020). We further showed that blocking NLRP3 inflammasome activation in the spinal cord significantly alleviated mechanical allodynia of CRPS-I model rats and reduced spinal glial cell over-activation, a critical step involved in central pain sensitization (Chen et al., 2020). These findings suggest spinal NLRP3 inflammasome as a key contributor to pain mechanism of CRPS-I. Therefore, we hypothesized that EA may intervene with NLRP3 inflammasome activation in spinal cord to exert analgesic effect on CRPS-I.

In this study, we employed the rat chronic post-ischemic pain (CPIP) model initially developed by Coderre et al. to mimic human CRPS-I (Coderre et al., 2004). We studied whether NLRP3 inflammasome is activated in the spinal cord of CPIP rats and whether EA intervention affects NLRP3 inflammasome activation. We further explored the cellular distribution of NLRP3 in spinal cord and examined whether EA affects NLRP3 activation in specific spinal cells. Finally, pharmacological methods that specifically targets against NLRP3 inflammasome were utilized to validate our findings. This study provides novel evidence showing that EA can attenuate NLRP3 inflammasome activation in spinal cord dorsal horn neurons, which in turn reduced spinal glial cell over-activation and contributes to EA's anti-allodynia on CRPS-I animal model.

## Methods and Materials

### Animals

Sprague–Dawley rats (male, 3–4 months of age) were bought from Shanghai Laboratory Animal Center of China. The animals were maintained in Zhejiang Chinese Medical University Laboratory Animal Center (5 animals/cage, 12 h dark-light cycle, 24 ± 2°C). Animals were provided with free access to food and water. All animals were given 1 week to accommodate to the new environment before any test. Animals were randomly allocated using random number table method. The group size was determined based upon our previous experience and studies using similar experimental protocols (Hu et al., 2020a; Yin et al., 2020). We also made sure that at least 5 or more samples were included for ANOVA comparison as recommended by statisticians (Arifin and Zahiruddin, 2017; Curtis et al., 2018). A total number of 193 rats were used in this study. The initial number of animals used per group was between 6 and 9. The specific number of animals included in each group is indicated in figures and figure legends.

### Research Reagents

NLPR3 specific antagonist MCC950 and the activator nigericin were purchased from APExBIO Technology (USA). IL-1 receptor antagonist IL-1Ra was purchased from Beyotime (China).

### Establishment of the Rat Model of CRPS-I

Chronic post-ischemia pain (CPIP) model was established *via* imposing ischemia and reperfusion to the hind limb of the rat as reported before (Coderre et al., 2004; Hu et al., 2020b; Nie et al., 2021). The rats were anesthetized by injecting 50 mg/kg (i.p.) sodium phenobarbital. A 7/32 (5 mm) internal diameter O-ring was used to ligate the hind limb at a position near the ankle joint for 3 h. After 3 h, the O-ring was removed. The control group of rats were treated with the same anesthetic steps but without O-ring ligature. No analgesic was provided during model establishment since it could feasibly interfere with the development of the pain state of this animal model (Li et al., 2022).

### Paw Swelling Measurement

The swelling of the hind paw was measured with a digital caliper as reported previously (Wang et al., 2020). The percent increase in paw thickness was used to indicate the paw edema. Three measurements were taken for each rat and the average value was obtained thereafter.

### Mechanical Allodynia

Mechanical allodynia was measured by methods described before (Chai et al., 2018). Each rat was allowed to acclimate to the testing conditions for 30 min beforehand. Mechanical allodynia was measured by applying von Frey filaments to the mid-plantar surface of the rat's hind paw. The “Up and Down” method was used for measurement and calculation of 50% paw withdrawal threshold as reported before (Dixon, 1980; Chaplan et al., 1994). The experimenter for behavioral tests was blinded to groupings.

### EA Intervention

The same EA procedure was adopted in the present study from our recent study, with some minor modifications (Hu et al., 2020a). Briefly, all animals included in this secession were immobilized gently with a self-made retainer. For EA treatment group, 4 acupuncture needle was inserted into bilateral ST36 and BL60 acupoints (with a depth of around 5 mm). The HANS-200A Acupoint Nerve Stimulator (Huawei Co., Ltd., China) was used to connect the acupuncture needles. A 2/100 Hz square wave with intensities from 0.5 to 1.5 mA was administered for 30 min per secession. For animals receiving sham EA intervention, the acupuncture needle was only inserted into bilateral BL60 and ST36 but no electrical stimulation was applied as reported (Liu et al., 2021). The EA or sham EA interventions were performed on animals on a daily basis for 7 days.

### Drug Preparation and Application

Specific NLRP3 inflammasome inhibitor MCC950 (APExBIO Technology, USA) was prepared in stock in DMSO and diluted in PBS prior to use. It was applied *via* intrathecal catheter (30 μg/rat/day, 25 μl injection volume). A vehicle containing (0.1% DMSO in PBS) was used as control. Nigericin, an NLRP3 activator (APExBIO Technology, USA), was dissolved in DMSO and further diluted in PBS. It was administered *via* intrathecal catheter (5 μg/rat/day, 25 μl injection volume) 45 min before EA treatment. Sham group rats received vehicle (PBS + 0.1% DMSO) injection. IL-1β receptor antagonist IL-1Ra (Beyotime, China) was dissolved in sterile saline solution, containing 0.1% DMSO. IL-1Ra was injected intrathecally at a concentration of 100 ng/rat/day once a day for 7 days (25 μl injection volume), whereas sterile saline containing 0.1% DMSO of an equal volume was used as vehicle injection. All injections were administered by a researcher not involved in behavioral testing.

### Lumbar Spinal Catheterization

The drug was applied *via* lumbar catheterization described in our previous study (Hu et al., 2020a). Briefly, rats were anesthetized with sodium pentobarbital (50 mg/kg, i.p.). The lumbar part was shaved and disinfected with 75% ethanol. A small incision was cut along L4–5 lumbar vertebrae. The intervertebral foramen was exposed after cutting intervertebral ligament. A PE-10 catheter prefilled with sterilized PBS was inserted into the subarachnoid space. The proper insertion of the catheter was indicated by tail-flick or paw retraction. After the insertion, the proper intraspinal location was examined by lidocaine (1%, 10 μl/rat) injection *via* catheter. The proper insertion of the catheter was demonstrated by a quick motor paralysis of the hind limbs, usually lasting 15–30 min. The catheter was secured and skin incision was sutured. The rats were then placed back into individual cages for recovery and under close monitoring.

### Western Blotting

The procedures were described in our previous study (Chen et al., 2020; Li et al., 2021). At Day 7, after the treatment procedure and behavioral test, the rats were deeply anesthetized with sodium pentobarbital (50 mg/kg, i.p.). The rats were then perfused transcardially through the aorta with 0.9% saline (4°C). Then the lumbar spinal cord tissues were collected quickly. The tissues were then homogenized in RIPA lysis buffer and centrifuged at 15,000 rpm for 15 min at 4°C. The supernatants were collected and measured the protein concentration using a BCA protein assay kit (Thermo Fisher, USA). Equal amounts of protein samples (10 μg) were electrophoresed on 5–12% SDS-polyacrylamide gels and transferred onto PVDF membranes (Bio-Rad, United States). After the membranes were blocked by 5% non-fat milk in TBST solution for 1 h at room temperature, the primary antibodies were applied overnight at 4°C and the HRP coupled secondary antibodies were applied for 2 h at room temperature. The antibodies used in the present study were listed as follows: IL-1β (1:500, rabbit polyclonal, #ab9722, Abcam), NLRP3 (1:500, rabbit polyclonal, #NBP2-12446, Novus), ASC (1:500, rabbit polyclonal, #PA5-88132, Thermo Fisher). Mouse anti-β-actin (HRP Conjugate, 1:5000, #ab20272, Abcam) was used as reference control. The expression levels of targeted protein are normalized to the density of β-actin.

### Immunofluorescence Staining

Procedures were described in our previous study (Liu et al., 2022). Briefly, embedded spinal cord samples were cut into frozen sections with the thickness of 20 μm. Then samples were mounted onto gelatin-coated glass slides for immunofluorescence staining. The sections were blocked in normal donkey serum (5%) in Tris buffered saline tween (TBST) for 1 h at room temperature, and then incubated with corresponding primary antibodies overnight at 4°C. The primary antibodies were as follows: mouse anti-GFAP (1:1000, #c9205, Sigma), mouse anti-OX42 (1:1000, #ab1211, Abcam), mouse anti-NeuN (1:1000, #ab104224, Abcam), and rabbit anti-NLRP3 (1:200, #PA5-79740, Invitrogen). Then the sample sections were labeled with the fluorescent secondary antibodies (Cy3-, Cy5-, or FITC-conjugated) for 1 h at room temperature after washing in the dark and counterstained with DAPI. The pictures of sample sections were captured by laser scanning confocal microscope (Nikon A1R, Japan). Images were captured with uniformed settings and experimenters were blinded to treatment groups for image quantification. Three to five random images from per rat tissue in each group were selected, averaged, and then compared as described previously (Liu et al., 2016; Li et al., 2021).

### Statistical Analysis

SPSS 19.0 (IBM Corp., USA) was used for statistical analysis. Data was presented as mean ± SEM. To compare data between 2 groups, the Student's *t*-test was used. To compare data among 3 or more groups, one-way or two-way ANOVA with Tukey's *post-hoc* test was applied. Statistical significance was accepted at a level of *p* < 0.05.

## Results

### The Establishment of the Rat CPIP Model and Nocifensive Behavioral Assessment

**Figure 1:**
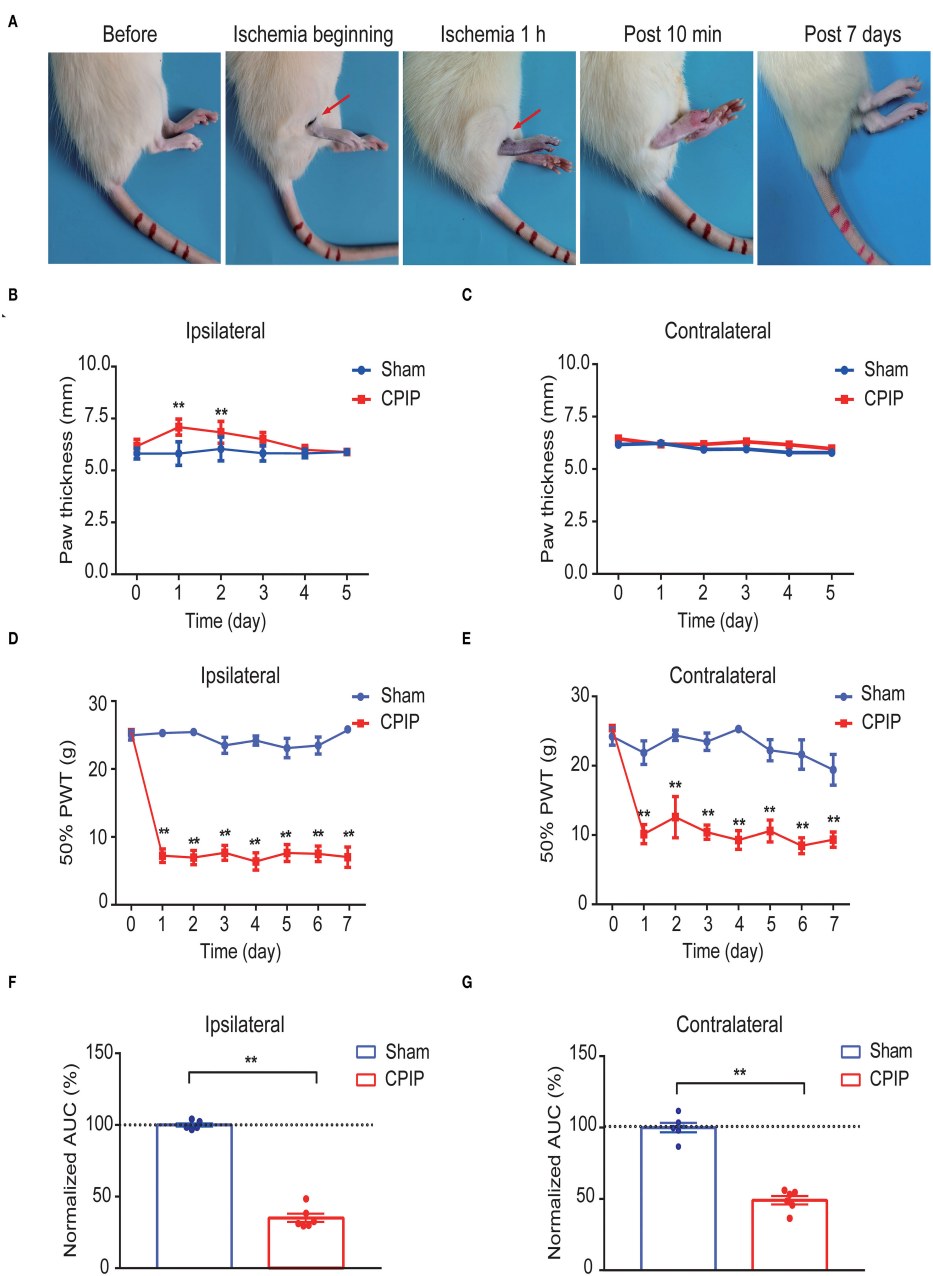
The establishment of the rat CPIP model to mimic CRPS-I and nocifensive behavior evaluation. **(A)** Representative pictures showing the changes of the hind paw after the O-ring ligation at different time points as indicated. The red arrow indicates the position of the O-ring. **(B,C)** Ipsilateral **(B)** or contralateral **(C)** hind paw swelling after model establishment. **(D,E)** 50% paw withdraw threshold of ipsilateral **(D)** or contralateral **(E)** hind paw from sham and CPIP model rats after model establishment. **(F,G)** Normalized area under the curve calculated from **(D)** and **(E)**, respectively. *n* = 6 rats/group. **p < 0.01 vs. Sham group. Two-way ANOVA with Tukey's *post-hoc* test was applied in **(B–E)**. One-way ANOVA with Tukey's *post-hoc* test was applied in **(F,G)**.

We first established the rat CPIP model to mimic human CRPS-I according to methods previously described (Coderre et al., 2004; Hu et al., 2020b). When the O-ring was placed, the hind paw displayed obvious swelling, accompanied with the appearance of cyanosis, a sign of tissue hypoxia (Figure 1A). The paw swelling lasted for 2 days and gradually returned to normal (Figures 1B,C). Moreover, the CPIP model rats displayed remarkable bilateral mechanical allodynia in the hind paws, which persisted over 7 days in our observation time frame (Figures 1D,E). AUC analysis indicated an overall pain response in the bilateral hind paws of the CPIP model rats (Figure 1F: *p* = 1.79 × 10^−9^ and Figure 1G: *p* = 4.82 × 10^−7^). These signs are consistent with previous studies (Coderre et al., 2004; Hu et al., 2019), demonstrating the successful establishment of the CPIP rat model.

### EA Effectively Ameliorated Mechanical Allodynia of CPIP Model Rats

**Figure 2:**
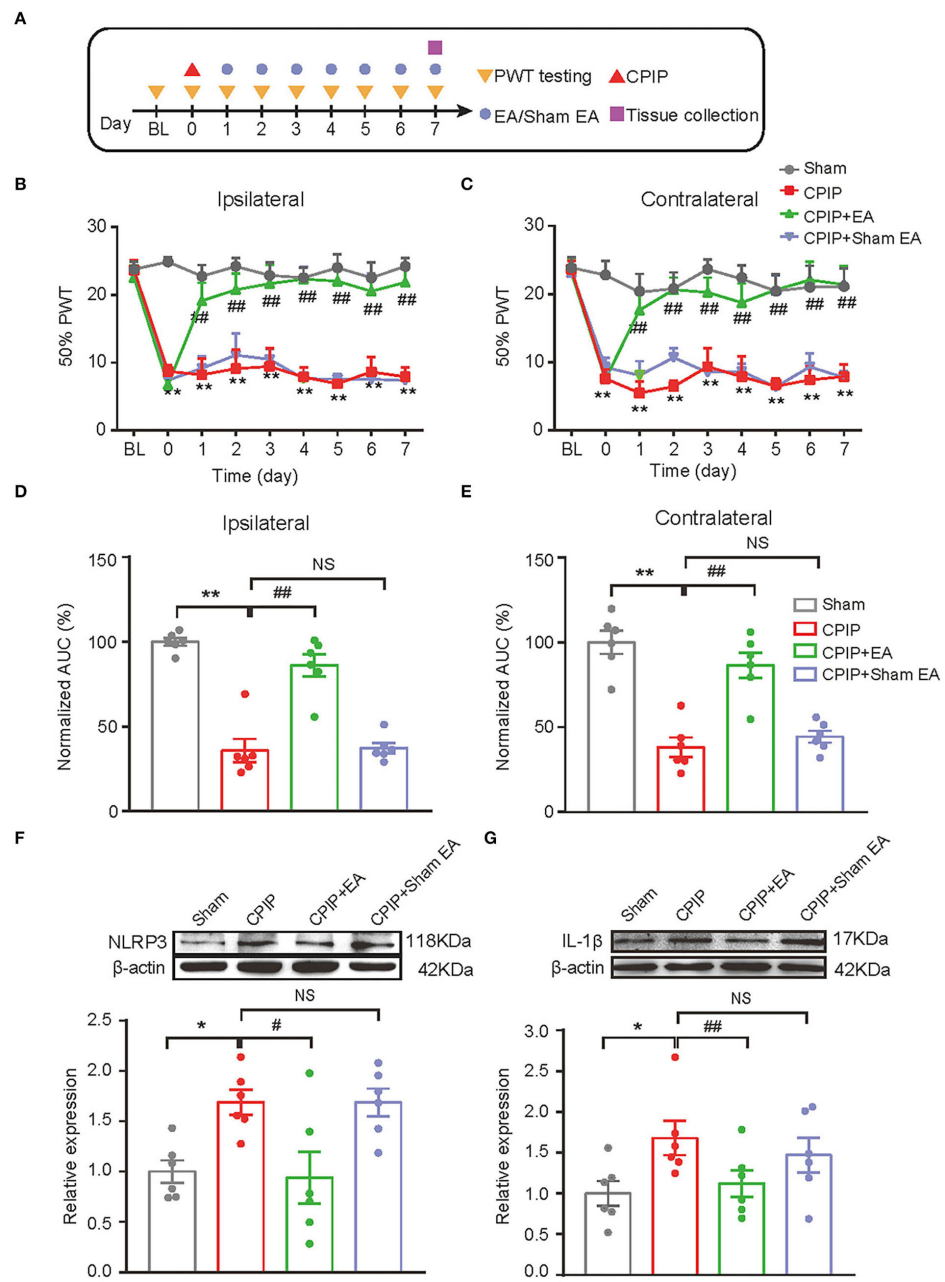
EA intervention attenuated mechanical allodynia of CPIP model rats and reduced NLRP3 inflammasome activation in spinal cord. **(A)** Experimental protocol showing time points for model establishment and EA intervention. **(B,C)** 50% PWT changes in ipsilateral **(B)** or contralateral **(C)** hind paw following EA/sham EA intervention. **(D,E)** Normalized AUC calculation from panel B&C. **(F,G)** Western blot showing protein expression of NLRP3 **(F)**, IL-1β **(G)** in ipsilateral spinal cord tissues. The upper panel shows representative Western blot images and the lower panel shows the summarized data. β-actin was used as a reference control. *n* = 6 rats/group. *p < 0.05 vs. Sham group. ^##^*p* < 0.01, ^#^*p* < 0.05 vs. CPIP group. NS, no significance vs. CPIP group. Two-way ANOVA with Tukey's *post-hoc* test was applied in panels B&C. One-way ANOVA with Tukey's *post-hoc* test was applied in **(D–G)**.

We started to investigate the therapeutic effects of EA on mechanical allodynia of the rat CPIP model. In our recent study, we identified 2/100 Hz as an optimal EA frequency for alleviating mechanical allodynia of CPIP rats (Hu et al., 2020a). Thus, in this study, we opt for 2/100 Hz as the frequency for EA intervention accordingly. Figure 2A illustrated the time schedule for EA/sham EA intervention. Daily EA intervention effectively reduced the mechanical allodynia of bilateral hind paws of CPIP rats (Figures 2B,C). Area under the curve (AUC) analysis showed an overall interventional effect of EA on mechanical allodynia of ipsilateral and contralateral hind paws (Figure 2D: *p* = 1.51 × 10^−7^, Sham vs. CPIP; *p* = 0.000006, CPIP vs. CPIP+EA; *p* = 0.997, CPIP vs. CPIP+shamEA and Figure 2E: *p* = 0.000003, Sham vs. CPIP; *p* = 0.000087, CPIP vs. CPIP+EA; *p* = 0.89, CPIP vs. CPIP+shamEA). In contrast, sham EA showed no obvious interventions (Figures 2B–E). This experiment corroborated our recent findings that 2/100 Hz EA is effective in alleviating mechanical allodynia of CPIP rats.

### EA Attenuated NLRP3 Inflammasome Activation as Well as Glial Cell Over-Activation in Ipsilateral Spinal Cord Dorsal Horn of CPIP Model Rats

In our recent study, we identified NLRP3 inflammasome in spinal cord dorsal horn (SCDH) as an important mechanism contributing to pain response of CPIP rats (Chen et al., 2020). NLRP3 inflammasome activation produced pro-inflammatory cytokine IL-1β in SCDH and triggers the activation of spinal astrocytes and microglia, which may result in central pain sensitization of CPIP model rats (Chen et al., 2018; Ji et al., 2019; Li et al., 2019). Therefore, we aimed to study whether EA intervention could affect NLRP3 inflammasome activation and spinal glial cell over-activation in SCDH of CPIP rats. We found that protein expression of NLRP3 and IL-1β were all significantly up-regulated in ipsilateral SCDH of CPIP group vs. sham group by Western blotting (Figure 2F: *p* = 0.04, Sham vs. CPIP; *p* = 0.0237, CPIP vs. CPIP+EA; *p* = 1.0, CPIP vs. CPIP+shamEA and Figure 2G: *p* = 0.018, Sham vs. CPIP; *p* = 0.048, CPIP vs. CPIP+EA; *p* = 0.439, CPIP vs. CPIP+shamEA). EA intervention significantly reduced the up-regulation of NLRP3 and IL-1β expression in ipsilateral SCDH, whereas sham EA had no such effect (Figures 2F,G). These data suggests that EA effectively attenuated NLRP3 inflammasome activation in SCDH of CPIP rats.

**Figure 3:**
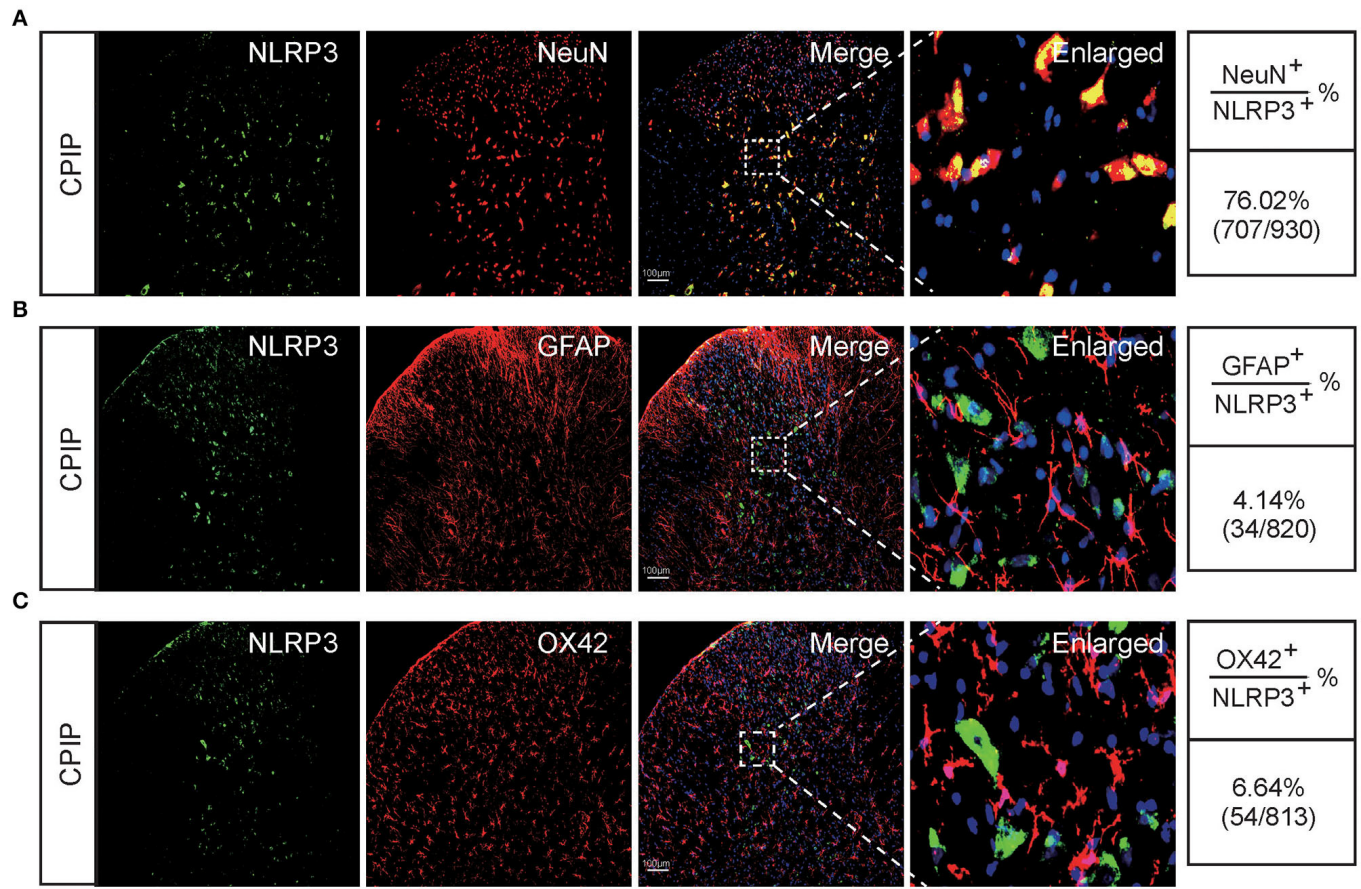
The cellular distribution of NLRP3 in spinal cord dorsal horn of CPIP model rats. **(A–C)** Double immunostaining showing NLRP3 (in green) co-expression with the neuronal marker NeuN (**A**, in red), astrocyte marker GFAP (**B**, in red) and microglial cell marker OX42 (**C**, in red) in ipsilateral spinal cord dorsal horn of CPIP rats. DAPI (in purple) was used as counterstain. Quantification is illustrated on the right of the panels. Scale bar indicates 100 μm. Data were obtained from 9 rats/group.

**Figure 4:**
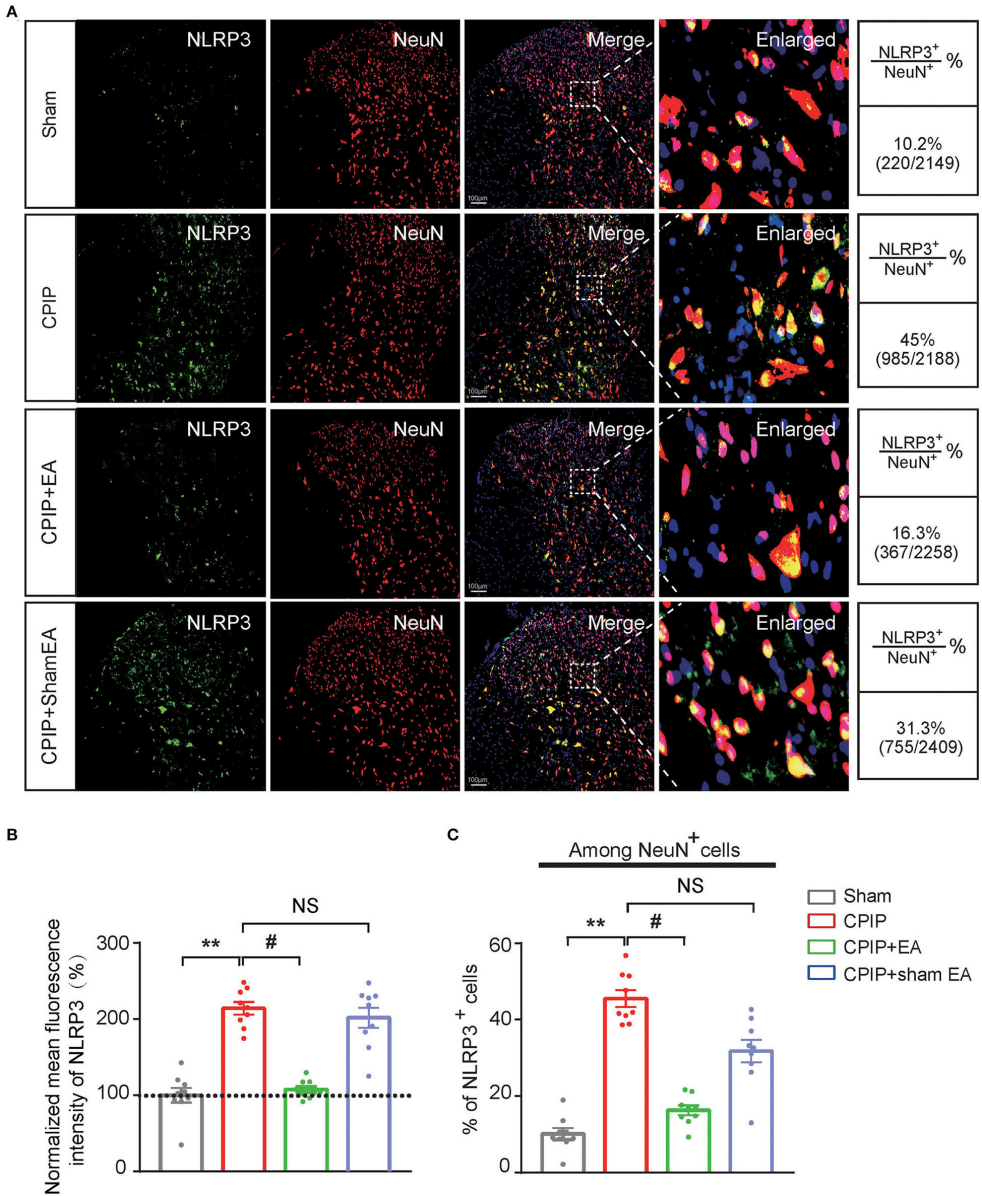
EA intervention attenuates NLRP3 overexpression in spinal cord dorsal horn neurons. **(A)** Double immunostaining showing NLRP3 (in green) co-expression with the neuronal marker NeuN (in red) in ipsilateral spinal cord dorsal horn of CPIP rats under different treatment conditions. DAPI (in purple) was used as counterstain. Quantification is illustrated on the right of the panels. **(B)** Summary of the mean fluorescence intensity of NLRP3. All intensities were normalized with the value of sham group. **(C)** Summary of the percentage of NLRP3+ cells among all NeuN+ cells. **p < 0.01 vs. Sham group. ^#^*p* < 0.05 vs. CPIP group. NS, no significance vs. CPIP group. Scale bar indicates 100 μm. Data were obtained from 9 rats/group. One-way ANOVA with Tukey's *post-hoc* test was applied in **(B,C)**.

**Figure 5:**
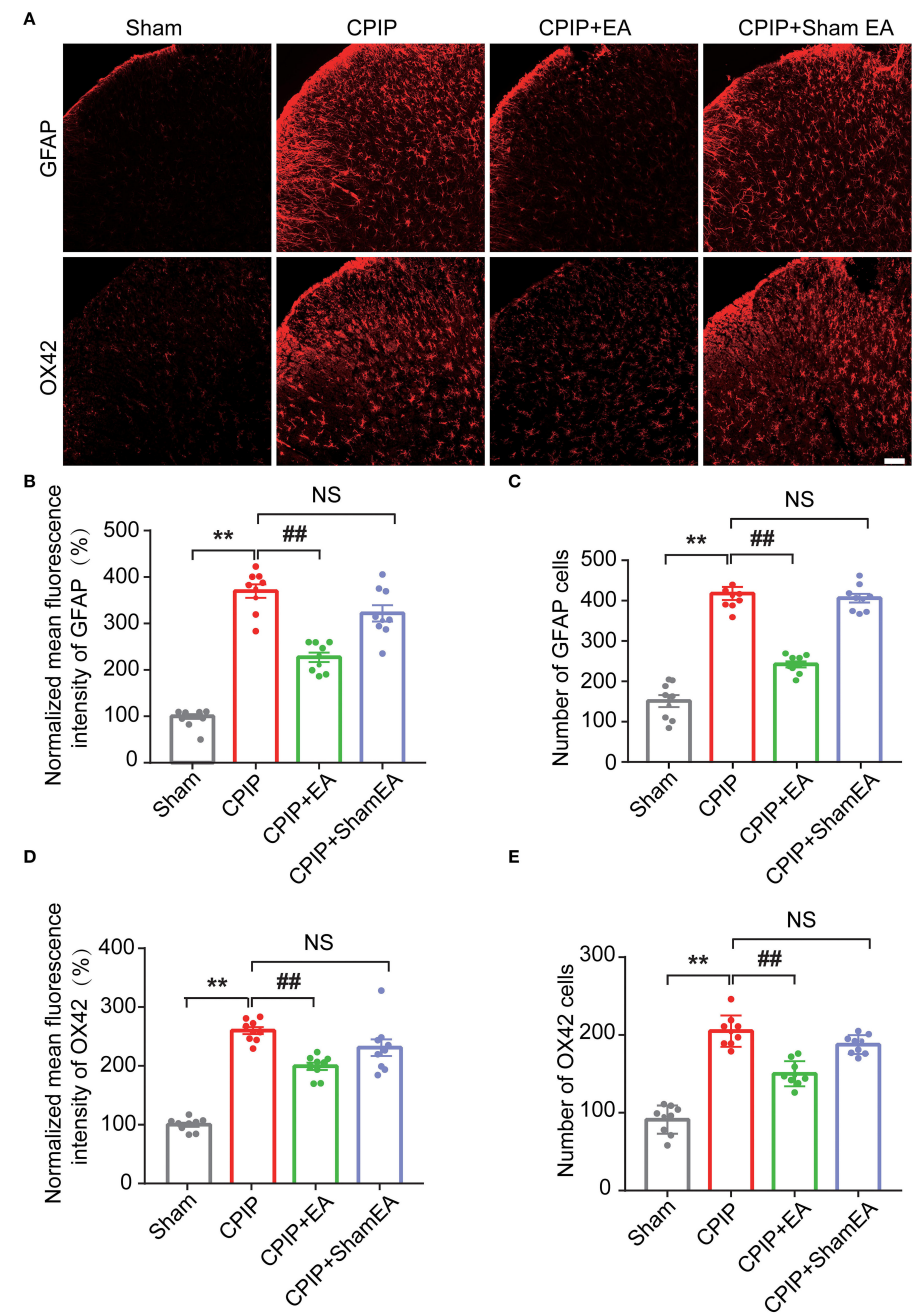
EA intervention reduces glial cell overactivation in spinal cord dorsal horn of CPIP rats. **(A)** Immunostaining pictures showing the effect of EA/sham EA intervention on astrocyte (Upper panels, marked with GFAP) and microglia (lower panels, marked with OX42) overactivation in SCDH. **(B)** Summary of the mean fluorescence intensity of GFAP, which were normalized with the value of sham group. **(C)** Summary of the total number of GFAP^+^ cells/observation field. **(D)** Summary of the normalized mean fluorescence intensity of OX42, which were normalized with the value of sham group. **(E)** Summary of the total number of OX42^+^ cells/observation field. Scale bar indicates 100 μm. Data were obtained from 9 rats/group. **p < 0.01 vs. Sham group. ^##^*p* < 0.01 vs. CPIP group. NS, no significance vs. CPIP group. One-way ANOVA with Tukey's *post-hoc* test was applied in **(B–E)**.

At this moment, the cellular distribution of NLRP3 in SCDH under CPIP condition still remains unknown. Therefore, in order to study the cellular distribution of NLRP3, we performed double immunostaining to co-label NLRP3 with markers for neuron (NeuN), astrocyte (GFAP) and microglia (OX42), respectively. As shown in Figures 3A–C, NLRP3 was predominantly co-expressed with NeuN (76.0%) in SCDH of CPIP rats. Only a few showed co-expression with GFAP (4.1%) or OX42 (6.6%). These results indicate that NLRP3 is exclusively expressed in SCDH neurons. Then we continued to explore whether EA intervention could suppress NLPR3 overexpression in SCDH neurons of CPIP rats. Immunostaining experiments indicated that EA significantly attenuated NLRP3 overexpression in SCDH of CPIP rats (Figures 4A,B). Moreover, EA-induced reduction of NLRP3 expression occurred largely in spinal cord neurons, indicated by the percentage of NLRP3 positive cells amongst NeuN positive cells (from 45% in CPIP group to 16.3% in CPIP+EA group, Figures 4A,C). In contrast, sham EA did not produce any such effect as EA (Figures 4A–C, Figure 4B: *p* = 4.86 × 10^−9^, Sham vs. CPIP; *p* = 2.19 × 10^−8^, CPIP vs. CPIP+EA; *p* = 0.789, CPIP vs. CPIP+shamEA; Figure 4C: *p* = 1.21 × 10^−12^, Sham vs. CPIP; *p* = 1.59 × 10^−10^, CPIP vs. CPIP+EA; *p* = 0.0624, CPIP vs. CPIP+shamEA). We recently found that NLRP3 inflammasome activation leads to spinal glial cell over-activation in CPIP animals, a critical step involved in central sensitization and chronic pain (Chen et al., 2020, 2021). Therefore, we proceeded to examine whether EA could attenuate spinal glial cell over-activation. As shown in Figures 5A–E, daily EA intervention significantly reduced spinal astrocyte as well as microglia over-activation in SCDH of CPIP rats, whereas sham EA did not produce such effect (Figure 5B: *p* = 7.04 × 10^−13^, Sham vs. CPIP; *p* = 2.42 × 10^−8^, CPIP vs. CPIP+EA; *p* = 0.053, CPIP vs. CPIP+shamEA; Figure 5C: *p* = 7.06 × 10^−13^, Sham vs. CPIP; *p* = 2.38 × 10^−10^, CPIP vs. CPIP+EA; *p* = 0.915, CPIP vs. CPIP+shamEA; Figure 5D: *p* = 7.81 × 10^−13^, Sham vs. CPIP; *p* = 0.0001, CPIP vs. CPIP+EA; *p* = 0.095, CPIP vs. CPIP+shamEA; Figure 5E: *p* = 7.12 × 10^−13^, Sham vs. CPIP; *p* = 4.99 × 10^−7^, CPIP vs. CPIP+EA; *p* = 0.151, CPIP vs. CPIP+shamEA).

### EA's Anti-allodynic Effect Is Mimicked by Pharmacological Blocking NLRP3 Inflammasome

**Figure 6:**
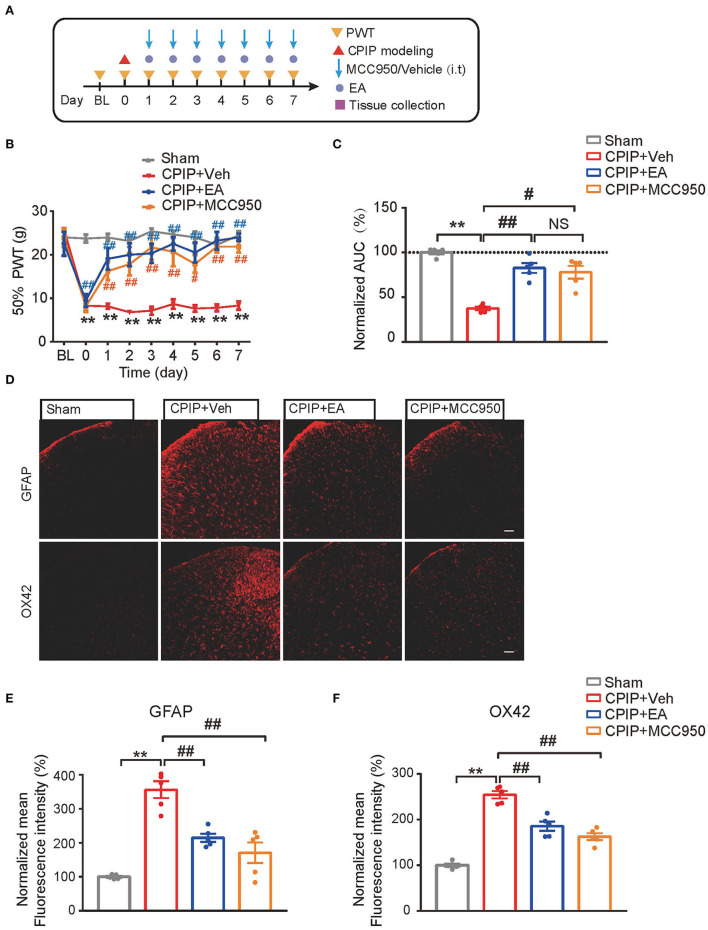
EA's anti-allodynic effect on CPIP rats is mimicked by pharmacological blocking NLRP3 inflammasome. **(A)** Experimental protocol. **(B)** 50% PWT following EA or MCC950 intervention (30 μg/rat/day, i.t.). **(C)** Normalized AUC deduced from **(B)**. **(D)** Immunostaining showing the effect of EA/MCC950 intervention on astrocyte (Upper panels, marked with GFAP) and microglia overactivation in SCDH. **(E,F)** Summary of the mean fluorescence intensity of GFAP **(E)** and OX42 **(F)**, normalized with the value of sham group. *n* = 5 rats/group. Scale bar indicates 100 μm. **p < 0.01 ^#^*p* < 0.05, ^##^*p* < 0.01. Two-way ANOVA with Tukey's *post-hoc* test was applied in panel B. One-way ANOVA with Tukey's *post-hoc* test was applied in **(C,E,F)**. NS, no significance.

MCC950 is a specific antagonist of NLRP3 inflammasome (Coll et al., 2015). In our recent study, we found intrathecal MCC950 treatment effectively attenuated NLRP3 inflammasome activation in SCDH and reduced mechanical allodynia of CPIP rats (Chen et al., 2020). We then set to compare the effect of EA with MCC950 on mechanical pain alleviation of CPIP rats. Intrathecal MCC950 treatment (30 μg/rat/day) or EA was applied to rats at time points shown in Figure 6A. As shown in Figures 6B,C, EA produced similar degree of mechanical allodynia alleviation compared with intrathecal MCC950 (Figure 6C: *p* = 6.25 × 10^−8^, Sham vs. CPIP+Veh; *p* = 0.000004, CPIP+Veh vs. CPIP+EA; *p* = 0.393, CPIP+EA vs. CPIP+MCC950; *p* = 0.0002, CPIP+Veh vs. CPIP+MCC950). NLRP3 inflammasome is involved in spinal glial cell over-activation in CPIP model rats (Chen et al., 2020). Therefore, we proceeded to test whether EA could attenuate spinal glial cell over-activation in CPIP model rats similarly with MCC950. As shown in Figures 6D–F, EA significantly reduced both spinal astrocyte and microglia over-activation in SCDH of CPIP rats, in similar degree with intrathecal MCC950 treatment (Figure 6E: *p* = 9.49 × 10^−7^, Sham vs. CPIP+Veh; *p* = 0.00098, CPIP+Veh vs. CPIP+EA; *p* = 0.000054, CPIP +Veh vs. CPIP+MCC950; Figure 6F: p = 9.48 × 10^−10^, Sham vs. CPIP+Veh; *p* = 0.000052, CPIP+Veh vs. CPIP+EA; *p* = 0.000001, CPIP +Veh vs. CPIP+MCC950).

### NLRP3 Inflammasome Activation *per se* Causes Persistent Mechanical Allodynia and Spinal Glial Cell Over-Activation in Naïve Animals

**Figure 7:**
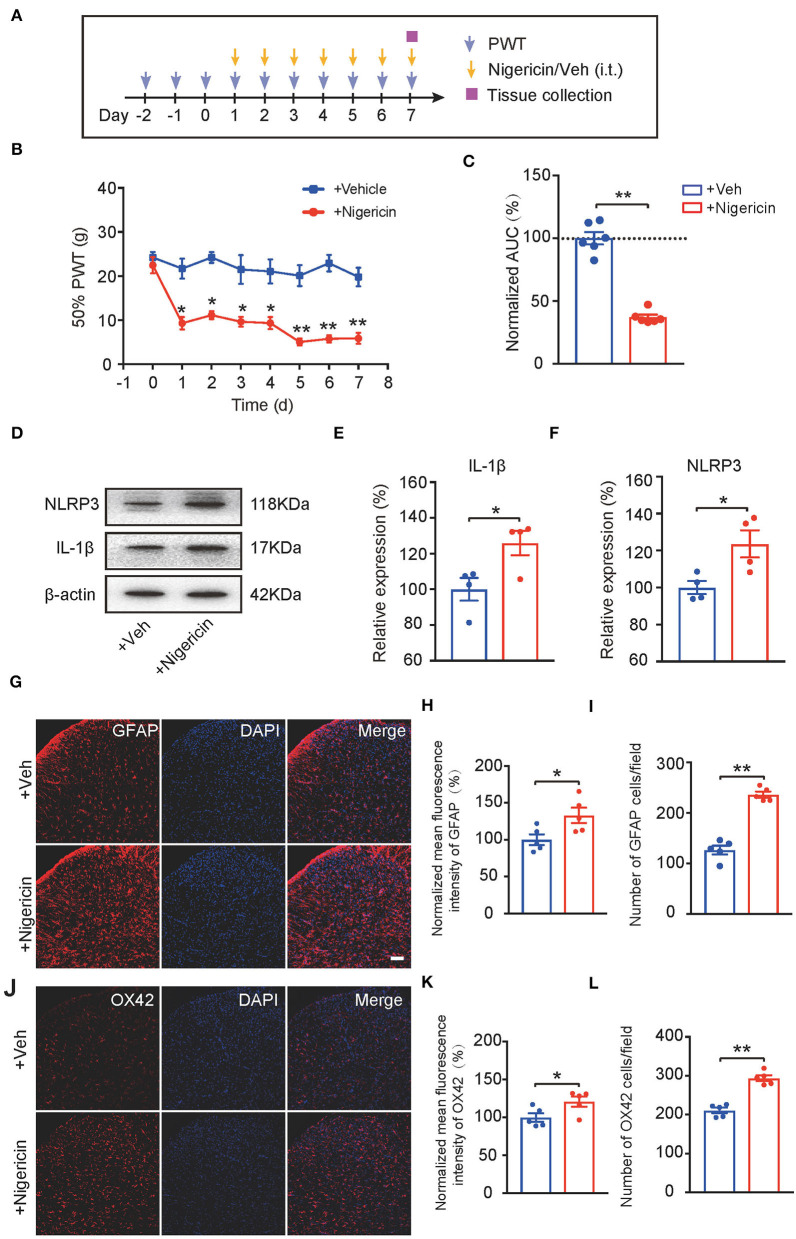
NLRP3 inflammasome activation *per se* causes mechanical allodynia and spinal glial activation in naïve animals. **(A)** Experimental protocol indicating treatment time points. **(B)** 50% PWT of rats after intrathecal vehicle (0.1% ethanol in PBS) or nigericin (5 μg/rat/day) injection. **(C)** Normalized AUC deduced from **(B)**. *n* = 6 rats/group. **(D–F)** Western blot showing protein expression of NLRP3 and IL-1β in spinal cord. **(D)** shows the representative images. **(E,F)** shows the summarized data of NLRP3 and IL-1β protein expression. **(G)** Immunostaining of GFAP after intrathecal nigericin in SCDH. **(H)** Summary of the mean fluorescence intensity of GFAP. **(I)** Summary of the total number of GFAP^+^ cells/observation field. **(J)** Immunostaining of OX42 after intrathecal nigericin in SCDH. **(K)** Summary of the mean fluorescence intensity of OX42. **(L)** Summary of the total number of OX42^+^ cells/observation field. Scale bar indicates 100 μm. *p < 0.05, **p < 0.01 vs. +Veh group. *n* = 4–6 rats/group. Two-way ANOVA with Tukey's *post-hoc* test was applied in panel B. Student's *t*-test was used in other panels.

Up-to-date, it remains elusive whether NLRP3 inflammasome activation *per se* can produce pain in naïve animals. In order to address this important issue, the NLRP3 inflammasome activator nigericin (5 μg/rat/day) was applied daily *via* intrathecal catheter to naïve rats (Mariathasan et al., 2006). Control rats received corresponding vehicle treatment (Figure 7A). Repetitive intrathecal nigericin administration produced robust and persistent mechanical allodynia among naïve rats compared with rats receiving vehicle treatment (Figures 7B,C, Figure 7C: *p* = 3.44 × 10^−7^). Intrathecal nigericin application produced obvious NLRP3 activation as well as IL-1β overproduction in spinal cord, as tested by Western blotting, which confirmed the efficacy of nigericin on NLRP3 inflammasome (Figures 7D–F, Figure 7E: *p* = 0.031; Figure 7F: *p* = 0.04). Furthermore, repetitively spinal nigericin administration triggers significant astrocyte and microglial activation in SCDH (Figures 7G–L, Figure 7H: *p* = 0.031; Figure 7I: *p* = 0.000006; Figure 7K: *p* = 0.042; Figure 7L: *p* = 0.000042). These data suggest that NLRP3 inflammasome activation *per se* can cause mechanical allodynia and produce spinal glial over-activation in naïve animals.

### Exogenously Applied NLRP3 Inflammasome Activator Nigericin Reversed EA-Induced Anti-allodynia in CPIP Rats

**Figure 8:**
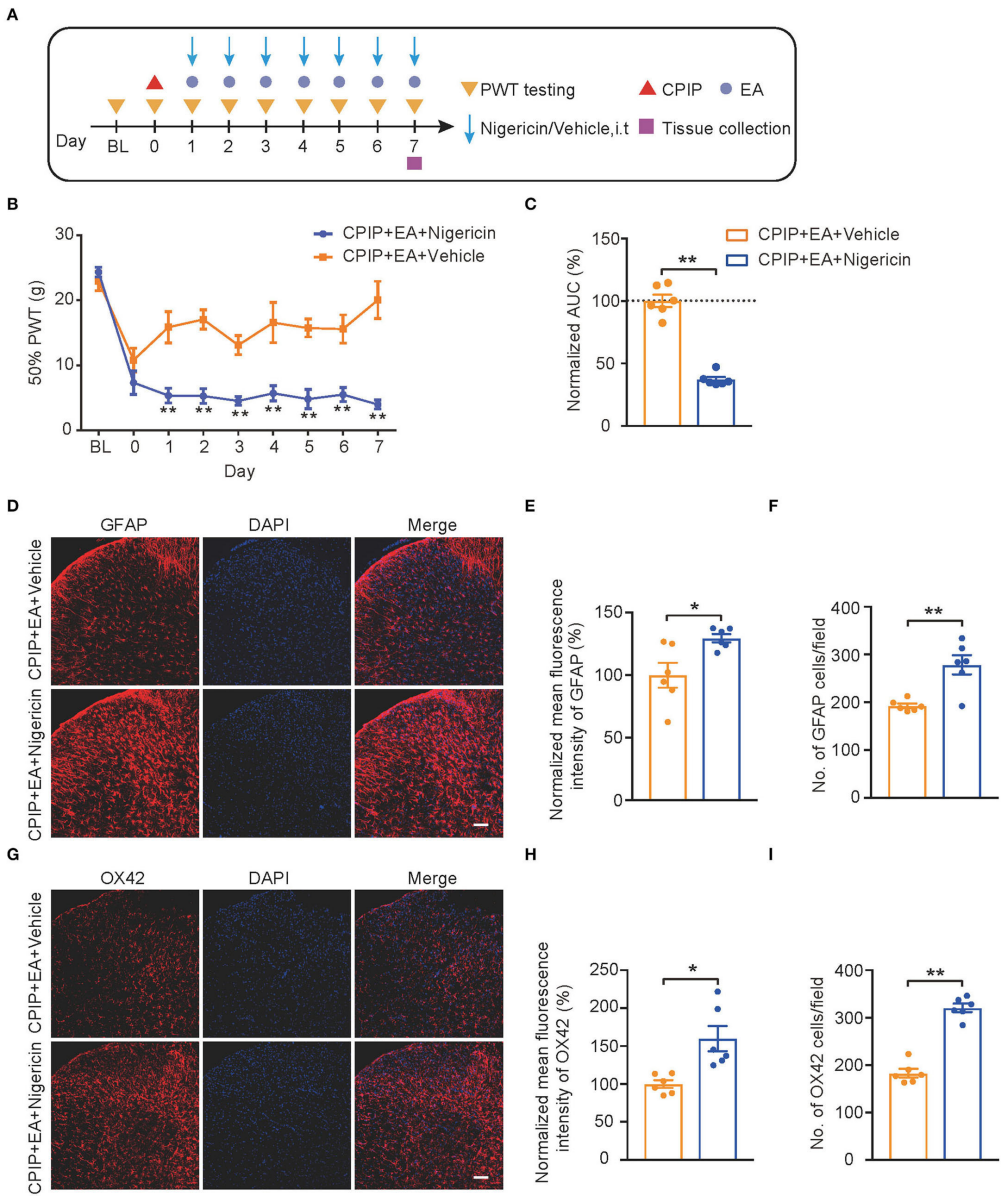
EA-induced anti-allodynic effect in CPIP rats is reversed by NLRP3 inflammasome activator nigericin. **(A)** Experimental protocol. **(B)** Time course showing the effect of intrathecal nigericin on EA-induced anti-allodynia in CPIP rats. **(C)** Normalized AUC deduced from **(B)**. **(D)** Immunostaining of GFAP in ipsilateral SCDH after intrathecal nigericin/Veh injection. **(E)** Summary of the mean fluorescence intensity of GFAP. **(F)** Summary of the total number of GFAP^+^ cells/observation field. **(G)** Immunostaining of OX42 in ipsilateral SCDH after intrathecal nigericin/Veh injection. **(H)** Summary of the mean fluorescence intensity of OX42. **(I)** Summary of the total number of OX42^+^ cells/observation field. Scale bar indicates 100 μm. *p < 0.05, **p < 0.01 vs. CPIP+EA+Veh group. *n* = 6 rats/group. Two-way ANOVA with Tukey's *post-hoc* test was applied in panel B. Student's *t*-test was used in other panels.

We then evaluated the impact of nigericin on EA-induced anti-allodynia in CPIP rats. As illustrated in Figure 8A, nigericin (5 μg/rat/day) or corresponding vehicle (0.1% ethanol in PBS, Veh) was applied *via* intrathecal catheter to CPIP rats 45 min before EA intervention. Daily intrathecal nigericin application significantly and persistently reversed the anti-allodynia effects of EA, as shown by comparison between CPIP+ EA+Nigericin and CPIP+EA+Vehicle group (Figures 8B,C, Figure 8C: *p* = 0.000065). Nigericin further reversed the effect of EA on spinal astrocyte and microglia over-activation (Figures 8D–I, Figure 8E: *p* = 0.017; Figure 8F: *p* = 0.0019; Figure 8H: *p* = 0.018; Figure 8I: *p* = 7.79 × 10^−7^). These results demonstrate that exogenously administered NLRP3 inflammasome activator nigericin reversed EA-induced anti-allodynia on CPIP rats.

### Spinal Blocking IL-1β Prevents Mechanical Allodynia and Spinal Glial Cell Over-Activation in CPIP Rats

**Figure 9:**
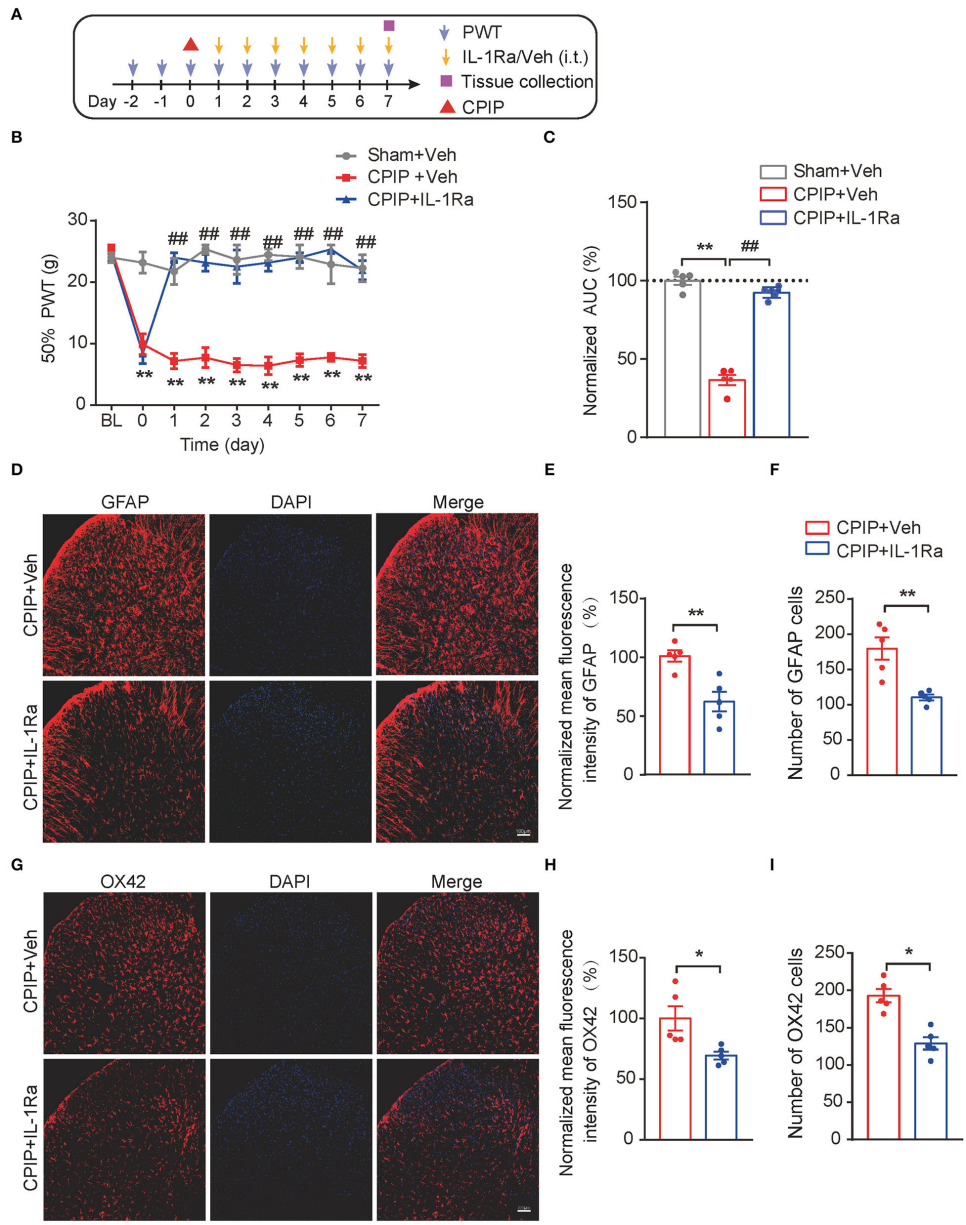
Spinal blocking IL-1β prevents mechanical allodynia and attenuates spinal glial over-activation in CPIP model rats. **(A)** Experimental protocol. **(B)** Time course showing the effect of intrathecal IL-1Ra (100 ng/rat/day) or vehicle (Veh 0.1% BSA in PBS) on mechanical allodynia of CPIP rats. **(C)** Normalized AUC deduced from panel B. **p < 0.01 vs. Sham group. ^##^*p* < 0.01 vs. CPIP+Veh group. **(D)** Immunostaining of GFAP in ipsilateral SCDH after intrathecal IL-1Ra/Vehicle injection. **(E)** Summary of mean fluorescence intensity of GFAP (normalized to CPIP+Veh group). **(F)** Summary of total number of GFAP^+^ cells/observation field. **(G)** Immunostaining of OX42 in ipsilateral SCDH after intrathecal IL-1Ra/Veh injection. **(H)** Summary of mean fluorescence intensity of OX42 (normalized to CPIP+Veh group). **(I)** Summary of total number of OX42^+^ cells/observation field. *p < 0.05, **p < 0.01 vs. CPIP +Veh group. *n* = 5 rats/group. Scale bar indicates 100 μm. Two-way ANOVA with Tukey's *post-hoc* test was applied in panel B. Student's *t* test was used in other panels.

The above results indicated that both MCC950 and EA could target on spinal NLRP3 inflammasome to reduce excessive production of IL-1β, a pro-inflammatory cytokine involved in pain mechanism. We reasoned that the excessive IL-1β production in the spinal cord may contribute to mechanical allodynia of CPIP rats. To further test this hypothesis, the IL-1 receptor antagonist IL-1Ra was intrathecally delivered *via* catheter to block IL-1β's effect in spinal cord (Figure 9A). IL-Ra treatment significantly attenuated mechanical allodynia of CPIP rats compared with vehicle-treated rats (Figures 9B,C, Figure 9C: *p* = 4.58 × 10^−10^, Sham+Veh vs. CPIP+Veh; *p* = 2.06 × 10^−9^, CPIP+Veh vs. CPIP+IL-1Ra). Moreover, IL-Ra treatment also reduced astrocyte and microglia over-activation in SCDH of CPIP rats (Figures 9D–I, Figure 9E: *p* = 0.0037; Figure 9F: *p* = 0.0031; Figure 9H: *p* = 0.02; Figure 9I: *p* = 0.0008). This result demonstrates a critical role of IL-1β in mediating spinal glial over-activation and mechanical allodynia of CPIP rats.

## Discussion

In the present study, we successfully established the rat CPIP model that mimics human CRPS-I. We found that the CPIP model rats developed robust bilateral mechanical allodynia, which could be relieved by daily EA intervention. NLRP3 inflammasome was activated in the spinal cord of CPIP rats, accompanied with the over-production of pro-inflammatory cytokine IL-1β. NLRP3 was predominantly expressed in SCDH neurons. EA intervention significantly reduced NLRP3 expression in SCDH neurons and further attenuated spinal glial cell over-activation. Moreover, the anti-allodynic effect, along with the attenuation of spinal glial cell over-activation produced by EA, were all mimicked by blocking NLRP3 inflammasome, whereas reversed by activating NLRP3 inflammasome *via* pharmacological interventions intrathecally. Finally, blocking IL-1β in spinal cord attenuated mechanical allodynia and spinal glial cell over-activation in CPIP rats. These results demonstrate that EA ameliorates mechanical allodynia of a rat model of CRPS-I through inhibiting NLRP3 inflammasome activation in spinal cord neurons.

The NLRP3 inflammasome is in charge of processing pro-inflammatory cytokine IL-1β and has been found to be involved in many inflammatory and pathological conditions (Mariathasan et al., 2006; Feng H. et al., 2020; Tong et al., 2020). Growing evidence has suggested that NLRP3 is dysregulated during chronic pain and participate in chronic pain mechanisms (Starobova et al., 2020; Chen et al., 2021). More recently, by means of RNA-Seq and functional validations, our group found that NLRP3 inflammasome expression (including NLRP3, Caspase-1 and IL-1β) is up-regulated in SCDH of CPIP model rats. Furthermore, pharmacological blocking NLRP3 inflammasome by specific antagonist MCC950 reduced mechanical allodynia of CPIP rats, as well as IL-1β over-expression and spinal glial cell over-activation (Chen et al., 2020). These results suggest that NLRP3 inflammasome is involved in IL-1β over-expression and spinal glial cell over-activation in CPIP model rats. However, the exact cellular distribution of NLRP3 and how NLRP3 may regulate spinal glial cells in CPIP condition still remain unknown. In the present study, our double immunostaining results identified that NLRP3 is predominantly expressed in spinal neurons, with only a few expressed in spinal glial cells. This result is consistent with a recent study showing that NLRP3 is exclusively distributed in spinal neurons of a rat model of bone-induced cancer pain (Chen et al., 2019). Therefore, it is possible that IL-1β produced from spinal neurons *via* NLRP3 inflammasome may act upon spinal astrocyte and microglia *via* neuro-glia crosstalk mechanism to trigger their over-activation, which in turn contributes to central sensitization and maintains chronic pain condition.

Pharmacological blocking NLRP3 results in pain relief in CPIP model rats and several other pain models (Chen et al., 2019, 2020; Huang et al., 2020). But it still remains unknown whether NLRP3 inflammasome activation *per se* is sufficient to induce pain. It remains possible that NLRP3 inflammasome needs to corporate with other machineries or mechanisms to produce chronic pain. This is an important question given the fact that more and more studies focusing on NLRP3 inflammasome's role in chronic pain have emerged recently (Chen et al., 2021). In this study, we solved this question by testing the direct effect of the compound nigericin, an NLRP3 inflammasome activator, on naïve animals. We found that intrathecal application of nigericin produced obvious NLRP3 activation and IL-1β overproduction in spinal cord tissues, which confirmed its activating effect on NLRP3 inflammasome. More importantly, intrathecal nigericin produced robust mechanical allodynia as well as spinal glial cell overactivation among naïve rats. Although further studies using NLRP3 knockout animals are needed for validating nigericin's effect, yet these experiments showed for the first time that NLRP3 inflammasome activation *per se* may possibly be able to induce pain.

Chronic pain is usually accompanied with the overexpression of a variety of inflammatory mediators (such as pro-inflammatory cytokines) in local inflamed tissues, peripheral nerves, as well as spinal cord tissues (Liu and Zhang, 2009; Liu et al., 2010; Ji et al., 2014; Jiang et al., 2020). These mediators make important contributions to the initiation and maintenance of chronic pain. Among these cytokines, IL-1β is an extensively studied cytokine. The excessive production of IL-1β is usually implicated in the etiology of chronic pain (Ren and Torres, 2009; Dinarello et al., 2012). IL-1β can either directly excite peripheral nociceptors to produce pain signal or may participate in peripheral or central pain sensitization via sensitizing nociceptors or facilitating neuron-glia crosstalk (Binshtok et al., 2008). Blocking IL-1β signaling has been verified to be an effective strategy for chronic pain treatment in both preclinical animal models and patients (Dinarello et al., 2012; Helyes et al., 2019). In the present study, we found that IL-1β expression is significantly up-regulated in spinal cord of CPIP rats. Pharmacological blocking IL-1β receptor by intrathecal IL-1Ra results in alleviation of mechanical allodynia of CPIP rats. Moreover, intrathecal IL-1Ra also reduced spinal astrocyte and microglial cell over-activation. These results indicate that IL-1β produced from NLRP3 inflammasome activation in SCDH neurons can trigger spinal glial over-activation and contribute to mechanical allodynia of CPIP model animals *via* neuron-glia crosstalk.

Electroacupuncture is an effective therapy with a few side effect for CRPS-I in clinic (Wei et al., 2019). However, the underlying mechanisms of EA against CRPS-I still remain largely unknown. Our recent study screened the EA frequency and identified 2/100 Hz as an optimal EA frequency for alleviating mechanical allodynia of CPIP rats (Hu et al., 2020a). We thus used 2/100 Hz EA in the present study and set to explore the anti-allodynia mechanism of EA on CRPS-I. We found that EA significantly reduced the overexpression of NLRP3 and IL-1β in spinal cord of CPIP rats. This result is a reminiscent of two recent studies reporting that EA significantly reduces NLRP3 inflammasome activation and IL-1β overproduction in skin tissues of CFA-induced inflammatory pain rats (Gao et al., 2018; Yu et al., 2020). However, it remains unexplored whether NLRP3 inflammasome activation actually contributes to the pain mechanism of these pain model animals. Therefore, in this study, we first performed a set of pharmacological experiments to confirm that spinal NLRP3 inflammasome activation indeed contributes to pain response as well as spinal glial cell overactivation in CPIP rats. We then demonstrated NLRP3 inflammasome activation *per se* can cause pain response in naïve animals. These results together confirmed the causal relationship between the pain response and NLRP3 inflammasome activation in spinal cord of CPIP rats in the first hand. Since we found that NLRP3 is predominantly expressed in neurons of SCDH, we then proceeded to examine whether EA was capable of reducing NLRP3 overexpression among these neurons in SCDH. Immunostaining revealed that EA attenuated NLRP3 overexpression exclusively in neurons of SCDH. The behavioral combined with pharmacological studies further demonstrated that EA-induced anti-allodynic effect was reversed by activating spinal NLRP3 inflammasome. Thus, our results demonstrate that EA reduces mechanical allodynia of CPIP rats *via* suppressing NLRP3 inflammasome activation in SCDH neurons.

## Conclusion

Our study provides novel evidence showing that EA attenuates NLRP3 inflammasome activation in spinal cord neurons, which in turn reduces Il-1β production and spinal glial cell overactivation and contributes to EA's anti-allodynia on CRPS-I animal model. Our study supports that EA can be used as an alternative and effective treatment option for CRPS-I.

## Data Availability Statement

The original contributions presented in the study are included in the article/supplementary material, further inquiries can be directed to the corresponding author/s.

## Ethics Statement

The animal study was reviewed and approved by Animal Ethics Committee of Zhejiang Chinese Medical University.

## Author Contributions

YZ, RC, QH, JW, HN, CY, YL, HW, BoyuL, and YT: investigation and data curation. JuF and XS: methodology. XJ, JiF, and BoyiL: conceptualization. YZ, RC, and QH: writing–original draft. BoyiL: writing–review and editing. All authors contributed to the article and approved the manuscript.

## Funding

This project was supported by the National Natural Science Foundation of China (81873365 and 82105014), Zhejiang Provincial Natural Science Funds (LQ21H270004 and LR17H270001), research funds from Zhejiang Chinese Medical University (2021JKZDZC07, Q2019J01, KC201943, and 2021J07), and Construction Plan for the Inheritance Studio of Xiaoqing Jin Famous Chinese Medicine Expert in Zhejiang Province (GZS2021011). Contents are solely the responsibility of the authors and do not necessarily represent the official views of the funders.

## Conflict of Interest

The authors declare that the research was conducted in the absence of any commercial or financial relationships that could be construed as a potential conflict of interest.

## Publisher's Note

All claims expressed in this article are solely those of the authors and do not necessarily represent those of their affiliated organizations, or those of the publisher, the editors and the reviewers. Any product that may be evaluated in this article, or claim that may be made by its manufacturer, is not guaranteed or endorsed by the publisher.
